# Autoimmune polyglandular syndrome type 2 and autoimmune hepatitis with thymoma-associated myasthenia gravis: case report

**DOI:** 10.1186/s12902-020-0498-5

**Published:** 2020-04-07

**Authors:** Hidefumi Inaba, Hiroyuki Ariyasu, Hiroshi Iwakura, Chiaki Kurimoto, Yoko Ueda, Shinsuke Uraki, Ken Takeshima, Yasushi Furukawa, Shuhei Morita, Yoshiaki Nakayama, Takuya Ohashi, Hidefumi Ito, Yoshiharu Nishimura, Takashi Akamizu

**Affiliations:** 1 The First Department of Medicine, Wakayama Medical University, 811-1, Kimiidera, Wakayama, Japan; 2 Department of Neurology, Wakayama Medical University, 811-1, Kimiidera, Wakayama, Japan; 3 Thoracic and Cardiovascular Surgery, Wakayama Medical University, 811-1, Kimiidera, Wakayama, Japan

**Keywords:** Autoimmune polyglandular syndrome type 2 (APS-2), Autoimmune Addison’s disease, Hashimoto’s thyroiditis, Autoimmune hepatitis, Thymoma-associated myasthenia gravis, Bronchial asthma

## Abstract

**Background:**

Autoimmune polyglandular syndrome type 2 (APS-2) is a rare and complex clinical entity, and little is known about its etiology and progression.

**Case presentation:**

A 52-year-old woman with autoimmune hepatitis (AIH) and bronchial asthma was diagnosed with APS-2; autoimmune Addison’s disease (AD), and Hashimoto’s thyroiditis (HT), and she underwent prednisolone (PSL) treatment. Five months later, she presented ptosis and was diagnosed with thymoma-associated myasthenia gravis (MG). Thymectomy and PSL treatment with immuno-suppressants appeared to ameliorate MG, AD, AIH, HT, and bronchial asthma. HLA typing analysis revealed that the patient had susceptible HLA alleles to MG, AIH, and HT in a Japanese population.

**Conclusions:**

This case suggests common endocrinological and autoimmune aspects of APS-2 and AIH with thymoma-associated MG, which are considered to be extremely rare complications.

## Background

Autoimmune polyglandular syndrome type 2 (APS-2) is a rare complex clinical entity. It is defined as autoimmune Addison’s disease (AD) concomitant with autoimmune thyroid diseases such as Graves’ disease and Hashimoto’s thyroiditis (HT), and/or type 1 diabetes mellitus, in the absence of hypoparathyroidism [1]. The prevalence of APS-2 is 1.4–4.5 per 100,000 and it most commonly affects middle-aged women [1].

Myasthenia gravis (MG) is a neuromuscular junction disease that is mostly associated with autoimmune antibodies, such as anti-acetylcholine receptor (AChR)-antibody (Ab) [2, 3]. Autoimmune hepatitis (AIH) is characterized by autoimmunity to hepatocytes with increase of antinuclear antibody (ANA) [4]. Co-existence of MG and AIH is rare [3, 5]. Moreover, cases of APS-2 with thymoma-associated MG and AIH are extremely rare, and their common etiology has been unclear [1]. Here, we report a case of APS2 accompanied by thymoma-associated MG and AIH. We also examined the HLA of the patient, including disease-susceptible allele.

## Case presentation

A 52-year-woman was admitted to our hospital because of 3-month history of loss of appetite, fatigue, and 2-month history of pigmentation of the oral mucosa and the tongue. She had been treated for bronchial asthma for 9 years by budesonide formoterol fumarate hydrate inhalation and oral theophylline. She had also had been diagnosed with AIH and had been taking azathioprine (50 mg/day) for 5 years.

**Fig. 1:**
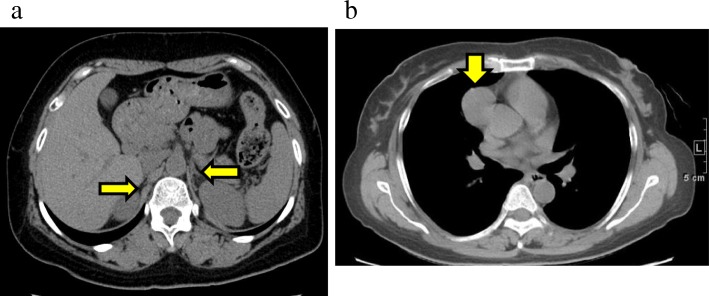
**a** On an abdominal CT, atrophy of the bilateral adrenal glands was seen (arrows). **b** A solid mass was seen in the anterior mediastinum (arrow) on a chest CT, suspected to be thymoma (34 × 34 mm in size)

She had no familial history of autoimmune disease. Her height was 144.5 cm, and body weight was 57.0 kg (BMI: 27.3 kg/m^2^). There was no apparent loss of body weight. Blood pressure was 88/63 mmHg. Her thyroid gland was firm and diffusely enlarged. In laboratory tests (Table 1), serum sodium and potassium levels were normal (under treatment of oral furosemide: 10 mg/day), and eosinophilia and hypoglycemia were not observed. In endocrinological examinations (Table 2), serum cortisol level at 06:00 was remarkably decreased, < 1.0 μg/dL (normal range: 2.9–19.4) and plasma adrenocorticotropic hormone (ACTH) level was elevated, 800.0 pg/mL (normal range: 7.2–63.3). Adrenal cortex autoantibodies (ACA) were positive, × 40 (normal range: < × 10). Abdominal CT showed that bilateral adrenal glands were slightly atrophied (Fig. 1a). Based on clinical findings and endocrinological data, she was diagnosed with autoimmune AD. She also had HT. Her anti-thyroglobulin antibody (TgAb) was elevated, 231.9 IU/mL (normal range: < 28.0) (Table 3). Ultrasonography examination showed diffusely enlarged thyroid gland, with coarse and hypoechogenic pattern. Both serum calcium level, 8.9 mg/dL (8.8–10.1), and plasma intact PTH level, 31 pg/dL (9.3–74.9) were normal (Tables 1, 2 and 3). Based on these findings, she was diagnosed with APS-2. In order to treat AIH and autoimmune AD, PSL was considered to be better than hydrocortisone. Therefore, PSL (10 mg/day) and fludrocortisone (0.1 mg/day) were started for autoimmune AD. The pigmentation of the oral mucosa and the tongue, the loss of appetite, the fatigue, and the hypotension disappeared within 3 months.

**Table 1:** Laboratory data on admission

	Results	Unit	Normal range
WBC	3330	/μL	(3500–9800)
(Neutro 51.1%, Eos 0%, Baso 0%, Lym 42.6%)			
Hb	12	g/dL	(12–15)
Plt	21.7 × 104	/μL	(13–37)
TP	6.2	g/dL	(6.7–8.1)
Alb	3.4	g/dL	(3.9–4.9)
AST	35	IU/L	(7–38)
ALT	14	IU/L	(4–44)
ALP	262	IU/L	(115–359)
G-GTP	19	IU/L	(9–35)
LDH	157	IU/L	(106–220)
BUN	13	mg/dL	(8–20)
Cr	0.53	mg/dL	(0.43–0.72)
T-bil	0.7	mg/dL	(0.2–1.2)
Ca	8.9	mg/dL	(8.8–10.1)
P	3.2	mg/dL	(2.7–4.6)
Na	137	mEq/L	(135–145)
K	3.7	mEq/L	(3.5–5.0)
Cl	101	mEq/L	(98–107)
PG	80	mg/dL	(70–109)
HbA1c	5	%	(4.6–6.2)
ANA	80	x	(< 40)
ds-DNA	negative		
RNP	negative		
Sm	negative		
SS-A	negative		
SS-B	negative		
Scl-70	negative		
C3	102	mg/dL	(65–135)
C4	19	mg/dL	(13–35)
Urinary analysis			
SG	1.02		
Protein	(−)		
Occult blood	(−)		
Sugar	(−)

**Table 2:** Endocrinological data on admission

	Results	Unit	Normal range
TSH	2.515	μIU/mL	(0.35–4.94)
FT3	3.51	pg/mL	(1.71–3.71)
FT4	0.93	ng/dL	(0.70–1.55)
TRAb	< 1.0	IU/mL	(< 2.0)
TgAb	231.9	IU/mL	(< 28.0)
TPOAb	< 5.0	U/mL	(< 16.0)
F	< 1.0	μg/dL	(2.9–19.4)
ACTH	800	pg/mL	(7.2–63.3)
PAC	1.2	ng/dL	(3.6–24)
PRA	14	ng/mL/H	(0.2–3.9)
i-PTH	31	pg/d	L (9.3–74.9)
ACA	40	x	(< 10)
GADAb	< 1.3	U/mL	(0–1.4)
IA-2Ab	negative		
Urinary examination			
free F	< 1	μg/day	(43–176)
GH	0.6	ng/mL	(0–2.1)
IGF-I	39	ng/mL	(78–213)
LH	5.7	mIU/mL	(1.7–11.2)
FSH	7.9	mIU/mL	(2.1–18.6)
E2	461	pg/mL	
PRL	38	ng/mL	(< 15)

**Table 3:** Results of HLA typing test of the patient

HLA	Patient allele	Susceptible autoimmune disease (References)
A	11:01/24:02	
B	39:01/51:01	
C	07:02/14:02	
DRB1	04:05/11:01	AIH Ref [4]. Other APS-2 case Ref [6]
DRB3	02:02	
DRB4	01:03	HT Ref [7], AIH Ref [4]
DQA1	03:03/05:05	
DQB1	03:01/04:01	AIH Ref [4], MG Ref [8]
DPA1	01:03/02:02	
DPB1	02:01/05:01	MG Ref [8]

Footnotes:

HLA-DRB1*04:05, DRB4, and DQB1*04:01 for AIH ref. [4], DRB4 for HT ref. [7], and DQB1*03 and DPB1*02:01 for MG ref. [8] were reported as disease-susceptible alleles

Konno, et al. reported a case of APS-2 similar to the current case; AD, HT and AIH with MG without thymoma and bronchial asthma who had HLA-A23, B52/62, and DR11/15 ref. [6]

**Fig. 2:**
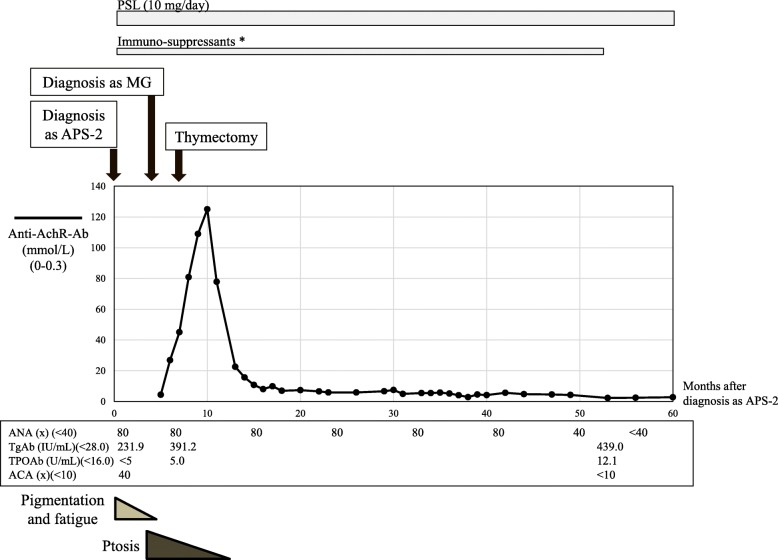
Clinical course after diagnosis of APS-2. Five months after this diagnosis, she was diagnosed with thymoma-associated MG. Anti-AchR-Ab titer gradually decreased. * Immuno-suppressants: Under treatment with azathioprine (50 mg/day), total thymectomy was conducted. After thymectomy, tacrolimus (3 mg/day) was started instead of azathioprine, and 25 months later, it was changed to cyclosporine (50 mg/day). Ten months later, the treatment was changed to ambenonium (5 mg/day), and ceased after 10 months (at 52 months)

Five months after diagnosis of APS-2, she noticed ptosis in both eyes which worsened in the evening. There was no muscle weakness, dysarthria, or dysphagia. She was readmitted to hospital. On readmission, anti-AChR-Ab level was increased, 4.4 nmol/L (normal range: 0–0.3). Anti-titin antibody was not measured. The repetitive nerve stimulation test at 3 Hz of the right facial nerve revealed decremental response. On chest CT, an anterior mediastinal mass (34 × 34 mm) was observed, which was suspected to be thymoma (Fig.1b). She was thus diagnosed with thymoma-associated ocular MG. Pyridostigmine treatment was begun, but due to adverse events including abdominal pain and skin rash, it was withdrawn. Since symptoms of the ocular MG were very slight, PSL (10 mg/day) for autoimmune AD and ocular MG was continued. Azathioprine (50 mg/day) for AIH was continued by the hepatologist. Subsequently, total thymectomy was conducted. The surgical specimen of mediastinal mass exhibited thymoma: histological type B2, pT2, R0. After the thymectomy, PSL and immuno-suppressant treatment was continued (Fig. 2). The level of anti-AchR-Ab for MG then decreased. Ptosis also improved within 5–6 months. After 42 months, immuno-suppressant treatment was ceased, but even after 10 months, MG had not worsened. Normalized ACA levels (< 10) at 53 months might suggest improvement of AD or atrophy of the adrenal glands. Although serum levels of TgAb and anti-thyroperoxidase antibody (TPOAb) increased during the course, thyroid function stayed normal during the entire course, thus HT had not worsened.

Since multiple concurrent autoimmune diseases were seen, the patient underwent HLA typing tests. She had HLA-A*11:01/24:02, B*39:01/51:01, C*07:02/14:02, DRB1*04:5/11:01, DRB3*02:02, DRB4*01:03, DQA1*03:03/05:05, DQB1*03:01/04:01, DPA1*01:03/02:02, and DPB1*02:01/05:01 (Table 3).

## Discussion and conclusion

APS is divided into 4 types, and APS-2 with autoimmune AD and HT is referred to as Schmidt’s syndrome [1]. Thymoma is reported in 15% of cases of MG, and half of cases of thymoma have concomitant MG [2, 6]. The prevalence of AIH in APS-2 is reported to be 4%, and MG in APS-2 is considered to be even more rare [3].

In the current case, based on the typical endocrinological findings, autoimmune AD and HT were diagnosed. The absence of hypoparathyroidism led to diagnosis of APS-2. Thymoma-associated MG concomitant with AIH and bronchial asthma was also observed. Although cases of APS-2 have been reported, to the best of our knowledge, cases of APS-2 with thymoma-associated MG are rare, and their complication with AIH and bronchial asthma has not been reported until now.

Multiple genetic and environmental factors seem to be associated with the development of APS-2, but its etiology is not understood [1]. In the current case, MG developed despite the patient receiving PSL and azathioprine therapy. The reason for this is unclear, but enlarged thymoma might affect the development of MG.

HLA has been reported to be a major genetic factor for APS-2. HLA-DR3/DR4 alleles or its haplotype are reported to be one of the large risk factors for APS-2 in Western people [1]. To date, however, predisposition of HLA alleles to APS-2 in Japanese has not been reported.

In Japanese cohorts, disease-predisposing alleles are as follows: HLA-DRB4 for HT [7], HLA-DRB1*04:05, DRB4 and DQB1*04:01 for AIH [4], and DQB1*03 and DPB1*02:01 for MG [8]. All of these alleles were seen in the current case. Konno, et al. reported a case of APS-2 similar to the current case; their patient had AD, HT and AIH with MG without thymoma and with no bronchial asthma but had HLA-A23, B52/62, and DR11/15 [9]. DR11 was also seen in the current case. Seker, et al. also reported a case of AD and MG with thymoma, but without HT or AIH [10]. They did not examine HLA typing. HLA typing test could be useful for patients with multiple autoimmune diseases to determine a common immunological factor. HLA typing test would reveal various disease-susceptible and disease-protective alleles. Thus, early prediction and intervention of the diseases may be possible, and such information could help patient care in the long-term.

Given that development of bronchial asthma is reported to be associated with HLA inheritance [11], concomitance of AD, HT, thymoma-associated MG, and possibly bronchial asthma, could be related to common HLA allele or haplotypes. Since autoantigen is presented on the surface of antigen-presenting cells with HLA-class I (cytotoxic effects of CD8+ T-cells) or HLA-class II (effector effects of CD4+ T-cells), common antigen may also be involved in multiple diseases. A common antigen among AD, HT, AIH, MG, and possibly bronchial asthma, may cause cross presentation and cross immuno-reactions, and this may in part explain the etiology in our case.

Interestingly, thymoma is often related to autoimmune diseases [2, 3, 9, 12]. In a long-term follow up (60 months) of 85 patients with thymoma, 47 patients had autoimmune diseases, 33 patients had MG, 4 patients had HT, and 1 patient had AIH [12].

Serum levels of TgAb and TPOAb were increased, in contrast with the decreased levels of anti-AchR-Ab during the course, suggesting that TgAb and TPOAb were not indicators for progression of HT [13]. Indeed, thyroid function had been normal during the long course, and HT did not worsen after thymectomy.

In conclusion, our long-term observation highlighted the autoimmune aspects of a patient with very rare concomitant presentation of APS-2 and AIH with thymoma-associated MG, and with bronchial asthma. Early steroid intervention and thymectomy seemed to be effective treatment. This case suggests common endocrinological and autoimmune aspects of APS-2 and AIH with thymoma-associated MG.

## Data Availability

All data related to this report are stored at Wakayama Medical University (Wakayama, Japan), and are available from the corresponding author on reasonable request.

## Abbreviations

ACA: Adrenal cortex autoantibodies
AchR: Acetylcholine receptor
ACTH: Adrenocorticotropic hormone
AD: Addison’s disease
AIH: Autoimmune hepatitis
ANA: Antinuclear antibody
APS-2: Autoimmune polyglandular syndrome type 2
HT: Hashimoto’s thyroiditis
MG: Myasthenia gravis
PSL: Prednisolone
TgAb: Anti-thyroglobulin antibody
TPOAb: Anti-thyroperoxidase antibody
